# Intractable hyponatremia complicated by a reset osmostat: a case report

**DOI:** 10.1186/s13256-022-03732-w

**Published:** 2023-01-14

**Authors:** Mohamed Hassan Kamel, Ashish Upadhyay, Steven C. Borkan

**Affiliations:** 1 Section of Nephrology, Department of Medicine, Boston Medical Center and Boston University School of Medicine, Boston, MA USA; 2 Evans Biomedical Research Center, 650 Albany St, Room 546, Boston, MA 02118-2518 USA

**Keywords:** Antidiuretic hormone, Reset osmostat, Vaptans, Primary polydipsia, SIADH

## Abstract

**Background:**

Hyponatremia associated with a low serum osmolality is a common and confounding electrolyte disorder. Correcting hyponatremia is also complicated, especially in the setting of chronic hyponatremia. Here, we provide a rational approach to accurately detecting and safely treating acute on chronic euvolemic hyponatremia in the setting of acute polydipsia with a chronic reset osmostat.

**Case presentation:**

A 71-year-old hispanic gentleman with chronic hyponatremia presented with hiccups, polydipsia, and a serum sodium concentration of 120 mEq/L associated with diffuse weakness, inattentiveness, and suicidal ideation. Symptomatic euvolemic hyponatremia warranted hypertonic saline treatment in the acute phase and water restriction in the chronic phase. Both interventions resulted in improvement in symptoms and/or the serum sodium concentration, but to a serum sodium level that persistently remained below the normal range. Remarkably, the urine osmolality appropriately fell when the serum sodium concentration fell below 126 mEq/L. Also remarkable was the appropriate increase in urine osmolality when the serum sodium concentration exceeded 126 mEq/L. The preservation of both concentration and dilution, albeit at a lower-than-normal serum osmolality, shows that the osmostat regulating antidiuretic hormone release had been “reset.” Both physiologic and pharmacologic resetting of the osmostat are discussed.

**Conclusions:**

Preservation of urinary concentrating and diluting ability at a lower-than-normal serum sodium concentration, especially in the setting of chronic hyponatremia, is diagnostic of a reset osmostat. The presence of a reset osmostat often confounds the treatment of concomitant acute hyponatremia. Early recognition of a reset osmostat avoids the need to normalize serum sodium concentration, expedites hospital discharge, and limits potential harm from overcorrecting acute hyponatremia.

## Introduction

Hyponatremia is a common disorder of water imbalance that is typically defined as a sodium concentration (Na) < 136 mmol/L [1]. Hyponatremia is not itself a disease, but rather a manifestation of pathological conditions that frequently pose diagnostic dilemmas partly based on the patient’s volume status [2]. Safe hyponatremia management vastly differs when the disorder is acute (< 48 hours duration) versus chronic (> 48 hours duration). Furthermore, acute overcorrection can cause devastating neurological sequelae including osmotic demyelination syndrome [2]. Balancing treatment of symptomatic hyponatremia with the risk of overcorrection requires a deliberate approach to both diagnosis and therapy. Appreciating that patients with chronic hyponatremia may also have a reset osmostat warrants assessment of both urine concentrating and diluting capacity, refines the target for hyponatremia correction, and promotes patient safety.

Here, we describe a case of acute, symptomatic hyponatremia complicated by persistent, euvolemic hyponatremia. Although the acute symptomatic hyponatremia associated with polydipsia before and during the hospital course was amenable to both water restriction and later, to hypertonic saline infusion, the serum sodium level remained lower than normal. At this depressed serum sodium level, our patient exhibited a normal capacity to dilute and concentrate his urine. These observations defined his chronic hyponatremia as being due to a reset osmostat, established a rational target for hyponatremia correction, and reduced the potential risk of osmotic demyelination caused by acute overcorrection of hyponatremia.

## Case presentation

A 71-year-old hispanic man with severe depression, type II diabetes mellitus complicated by gastroparesis and peripheral neuropathy, as well as chronic obstructive pulmonary disease, presented to the hospital with diffuse weakness, inattentiveness, and suicidal ideation. On admission, he described feeling unwell for the prior 3 weeks and admitted to daily consumption of more than 4 gal (> 15 L) of bottled water in addition to sugar-sweetened beverages (primarily soda). As a result of nausea, he stopped eating solid food 4–5 days prior to admission and also discontinued his home medications including metformin, glargine and lispro insulin, lisinopril, amlodipine, metoclopramide, pantoprazole, and trazodone. There was no family history of hyponatremia.

On physical examination, vital signs were notable for a temperature of 36.4 °C, heart rate of 94 beats/minute, blood pressure of 158/103 mmHg, respiratory rate of 18 breaths/minute, and a peripheral capillary oxygen saturation of 97% on room air. He was noted to be a well-developed man with truncal obesity. He exhibited a flat affect and had frequent hiccups. His cardiac examination was unremarkable; lung auscultation revealed decreased basilar inspiratory breath sounds and expiratory wheezing without a prolonged expiratory phase; neurological examination was unremarkable; axillary membranes were moist, capillary refill, skin turgor, and tearing were normal; his extremities were warm and well-perfused with normal pulses but without pretibial edema.

Initial laboratory investigations including serum (Table 1), urine, and endocrine (Table 2) studies were most striking for a low serum sodium concentration (Na) of 120 mEq/L associated with a serum osmolality of 263 mOsm/kg, a urine osmolality of 155 mOsm/L, and a urine sodium of 38 mEq/L. A chest radiograph, electrocardiogram (showing normal sinus rhythm a rate of 86 BPM) and computerized tomography (CT) study of the brain were unremarkable. Screening tests for thyroid and adrenal disorders were unremarkable.

**Table 1:** Admission serum studies

Parameter	Result	Reference range
Sodium, mEq/L	120	135–145
Potassium, mEq/L	4.2	3.5–5.1
Chloride, mEq/L	91	98–108
Carbon dioxide, mEq/L	26	20–31
BUN, mg/dL	9	8–20
Creatinine, mg/dL	0.82	0.5–0.9
Glucose, mg/dL	116	70–100
Calcium, mg/dL	9.4	8–10.5
Albumin, g/dL	4.0	3.5–5
Osmolality, mOsm/kg H_2_O	263	275–295
Uric acid, mg/dL	4.8	3.4–7.0

*BUN* Blood urea nitrogen

**Table 2:** Admisison urine and serum endocrine studies

Parameter	Result	Reference range
*Urine studies*		
Urine osmolality, mOsm/kg H_2_O	155	50–1200
Urine sodium, mmol/L	38	–
Urine potassium, mmol/L	27.2	–
*Endocrine studies*		
Morning cortisol, μg/dL	8.1	4–23
TSH, IU/mL	1.83	0.35–4.9

*TSH* thyroid stimulating hormone

Review of his early admission data revealed that the patient’s blood pressure was elevated (that is, not reduced as in patients with hemodynamically significant hypovolemia) and his heart rate remained below 100 bpm. The elevated blood pressure was associated with recent discontinuation of home antihypertensive medications due to nausea. His volume examination remained normal during the entire hospitalization and his chest radiographs showed no evidence of hypervolemia. His urine output consistently measured between 1.5 and 3.2 L per day. The admission urine sodium of 38 mEq/L did not suggest high renal sodium avidity.

**Fig. 1:**
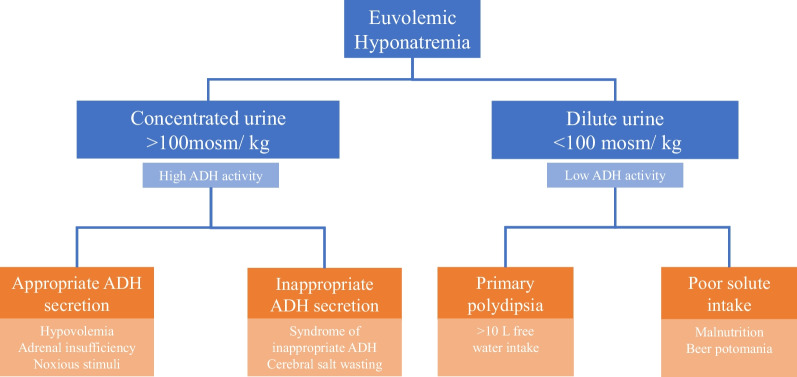
Euvolemic hyponatremia characterized by urine concentration. Urine osmolality serves as a surrogate marker for antidiuretic hormone activity in hyponatremic patients. The etiologies of hyponatremia are characterized by urine osmolality that reflects antidiuretic hormone activity

Due to his suicidal ideation and overt polydipsia, he was placed under direct, continuous supervision and fluid restriction was strictly enforced for the first 24 hours of admission. His Na improved but failed to normalize (Fig. 1). As his serum sodium concentration rose, the hiccups, altered sensorium, and anorexia improved. Although a minimally elevated osmolal gap (12 mOsm/kg) was detected on admission, the patient denied exposure to commonly encountered intoxicants (for example, ethanol, methanol, or ethylene glycol). Since the serum osmolality was not obtained until 2 hours after admission, we assumed that the unexpected osmolal gap measurement reflected a serum sodium concentration that had modestly increased above the initial value of 120 mEq/L (shown below).

Over the next 2 days, direct supervision was withdrawn, and fluid restriction was no longer reinforced. His serum sodium concentration again fell to 119 mEq/L and was associated with recurrent hiccups and new onset confusion. With worsening hyponatremia and hypo-osmolality, the patient’s urine osmolality fell to 88 mOsm/kg (that is, maximally dilute), indicating an intact urinary dilution system. To partially correct symptomatic hyponatremia, the patient received an intravenous bolus of 100 mL of 3% hypertonic saline over 1 hour, followed by 500 mL over the next 8 hours as calculated from the Androgue–Madias formula [3]. After his serum sodium concentration reached 126 mEq/L, both his confusion and hiccups abated, yet hyponatremia and hypo-osmolality persisted. The term “osmostat” refers to the regulatory neuronal osmoreceptor cells in the anterior hypothalamus that detect changes in blood osmolality, resulting in the either suppression or activation of antidiuretic hormone (ADH) release from neurosecretory cells. A reset osmostat (RO) is defined by an altered threshold for ADH release (either reduced or increased) associated with a serum sodium concentration that is persistently abnormal (also termed “chronic dysnatremia”). This definition perfectly fit our patient’s laboratory values.

## Discussion

**Fig. 2:**
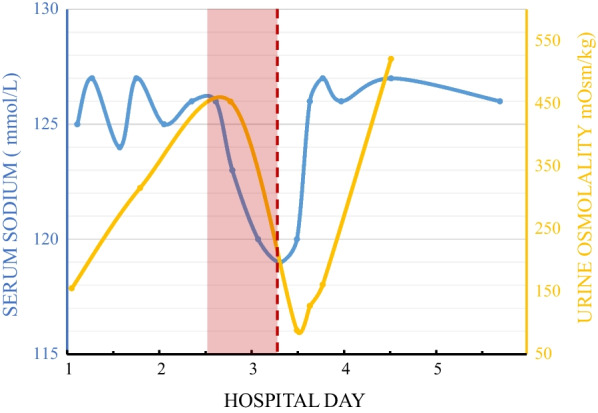
Urine osmolality in response to changes in serum sodium concentration throughout the hospital stay. Serum sodium (blue) fluctuates throughout hospital stay with an associated compensatory change in urine osmolality (yellow). During unsupervised time periods (shaded red), the patient had unlimited access to water. Hypertonic saline (dashed red line) was administered in response to symptomatic hyponatremia

Hyponatremia can be associated with low, normal or high intravascular volume status. In this case, the serum was clearly hypotonic (commensurate with a reduced serum sodium concentration) and the physical examination revealed clinical euvolemia suggesting “euvolemic hyponatremia.” Euvolemic hyponatremia signifies a disorder of renal diluting capacity that results from diverse pathologies. In patients with hypotonic hyponatremia, urine osmolality estimates ADH activity, and together with the history and volume assessment, directs the differential diagnosis (Fig. 2).

Our patient’s history was striking for both acute (in hospital) and chronic polydipsia of several months’ duration. However, his admission urine osmolality was 155 mEq/L (that is, not < 100 mOsm/L and therefore not maximally dilute). We hypothesized that the failure to achieve maximal urinary dilution was partly due to several days of poor dietary solute intake. This phenomenon has been reported in patients with the classic “tea-and-toast diet” and results in submaximal urinary dilution due to an inadequate solute intake that limits water excretion [4]. Our patient’s reduced dietary intake for several days prior to admission and a relatively low blood urea nitrogen (BUN) level both suggested that limited protein and solute intake contributed to his reduced ability to secrete water. However, his obese habitus [body mass index (BMI) 32], and normal serum albumin suggested a more robust, long-term nutritional status that would be adequate to support normal water excretion. Clinical history, vital signs, physical examination, and laboratory testing excluded potential contributions of abnormal thyroid and adrenal dysfunction to his hyponatremia. The combination of a normal uric acid level and relatively low urine osmolality were also atypical for the syndrome of inappropriate ADH (SIADH) secretion. Further, the family history was unremarkable for hyponatremia and therefore did not suggest nephrogenic syndrome of inappropriate antidiuresis [5].

A review of the patient’s electronic medical record revealed mild, chronic hyponatremia with baseline serum sodium values ranging between 123 and 133 mEq/L for at least 3 years prior to presentation. Upon further questioning, he endorsed chronic polydipsia that episodically worsened with either dyspepsia or anxiety. He also noted that increasing his water intake consistently stimulated recurrent hiccups and prompted additional water intake. The patient’s ability to both dilute and concentrate his urine and chronic hyponatremia suggested that a reset osmostat resulted in an abnormal threshold for ADH release as well as ADH suppression.

The patient was discharged on hospital day 6 with a stable serum sodium concentration of 133 mEq/L and was maintained on a daily fluid intake restricted to 1.8 L/day without salt tablets. At the time of discharge, he remained euvolemic, normotensive, and asymptomatic. Seven months later, he returned to the hospital with confusion, intractable hiccups, and a serum sodium of 117 mEq/L. Immediately prior to the second admission, he admitted to ingesting more than 6 L of water daily. Symptomatic hyponatremia again responded to 3% saline infusion and water restriction. Interestingly, ethanol itself is known cause of hiccups, most likely via stimulation of GABA receptors that increase efferent innervation of motor fibers of phrenic nerve that contract the diaphragm and accessory nerves that mediate intercostal muscles contraction [7]. Although it is unusual for hyponatremia itself to induce persistent hiccups [6], our patient later admitted that intermittent alcohol intoxication caused his hiccups and also stimulated his desire to “drink more water.” His discharge serum sodium concentration was again low at 131 mEq/L, suggesting that his osmostat remained reset at a lower than normal serum osmolality.

In the syndrome of inappropriate ADH (SIADH) caused by medications or other nonosmotic stimuli, patients exhibit persistent ADH secretion despite hypo-osmolality. In contrast, our patient discontinued his home medications prior to admission and showed wide fluctuations in urine osmolality (from < 100 to > 400 mOsm/kg) that would not be expected in the presence of an elevated ADH level. Poor dietary intake for several days prior to admission and a relatively low BUN level both supported limited protein and solute intake. However, his clinical history, obese habitus (BMI 32), and normal serum albumin suggested a normal level of nutrition. Clinical history, vital signs, physical examination, and laboratory testing excluded potential contributions of abnormal thyroid and adrenal dysfunction to his hyponatremia. The combination of a normal uric acid level and relatively low urine osmolality were also atypical for SIADH. Although not likely to be causal in our patient, osmoreceptors were recently detected in human gastrointestinal cells, suggesting that the brain is not the exclusive site for regulating thirst [8]. Curiously, these gastric receptors suppress thirst even before serum osmolality is altered by an oral water load, illustrating the exquisite regulation of water balance in the human body at multiple anatomic sites [9].

Although initially thought to be rare [10], a reset osmostat with sustained hyponatremia has been reported from a myriad of physiologic and pathologic processes. This condition is defined by in patients with persistent hyponatremia with hypo-osmolality but intact urinary concentrating and diluting capacity. Specifically, chronic hyponatremia due to a reset osmostat has been reported in patients with gastric carcinoma [11], tuberculosis [12], neurological disease [13, 14], and head trauma [10]. Interestingly, chronic exogenous desmopressin abuse also resets the osmostat and this abnormality can persist for several months or more [15]. A subset of patients with primary polydipsia (as detected in our patient), increase their urine concentration (that is, release ADH) during fluid deprivation, albeit at a lower than normal serum osmolality [16]. A reset osmostat also occurs in normal pregnancy but returns to normal postpartum [17], showing that the osmostat setpoint is remarkably flexible and can be reset when physiologic circumstances alter circulating blood volume.

As observed in our case, substantial evidence suggests that psychiatric patients with polydipsia reset their osmostat. In a series of 20 patients with psychogenic polydipsia and hyponatremia, moderate-to-severe hyponatremia (98–124 mEq/L), and an astonishing range of daily free water clearance between 12 and 36 L was observed after water intake of 7–43 L daily [16]. Remarkably, seven patients subjected to fluid restriction experienced a “reset osmostat” characterized by urinary osmolality that exceeded serum osmolality when serum osmolality rose only to 242–272 mOsm/kg, a level significantly below the normal range. Based on existing literature [16] and our patient’s ability to concentrate or dilute his urine at a lower serum sodium setpoint than expected, we speculate that chronic hyponatremia itself resets the osmostat by altering the feedback loop located in the hypothalamic-pituitary axis that controls thirst [18]. Taken together, these reports suggest that hypothalamic–pituitary axis may be amenable to pharmacologic intervention that resets the osmotic setpoint.

Unfortunately, no clear recommendations exist for treating patients with a reset osmostat, including those with concomitant psychogenic polydipsia with a predisposition to severe or symptomatic hyponatremia [19]. Although a reset osmostat might seem innocuous, substantial evidence suggests that mild hyponatremia (< 135 mEq/L) impairs balance and gait [20], increases fall risk [21], short-term memory loss [22], and also promotes progressive osteoporosis [23] as well as fractures, especially in the elderly [24]. A number of interventions have been proposed to address this clinical challenge. Independent of an antipsychotic effect, clozapine suppresses thirst in patients with primary polydipsia [19, 25, 26]. However, it is unclear whether suppressing primary polydipsia actually resets an abnormal osmostat [19, 25, 26]. Due to their potent ADH effects, it is tempting to suggest that ADH antagonists that pharmacologically increase Na might reset the osmostat to a normal setpoint [27]. If so, then resetting the osmostat in patients with chronic hyponatremia might be a novel indication for these agents. On the other hand, the salt tablets, a maneuver that increases the serum sodium concentration by only 2–3 mEq/L [28], can also increase thirst in patients predisposed to polydipsia and therefore risks inadvertent worsening of hyponatremia.

## Conclusions

In summary, a reset osmostat is a complex pathophysiological condition that complicates the assessment and treatment of persistent hyponatremia. Despite substantial evidence of toxicity resulting from mild, chronic hyponatremia, no clear guidelines exist for managing a reset osmostat. In patients with underlying primary polydipsia and psychiatric illness, antipsychotic medications and water restriction can improve serum sodium control. Clinical trials are needed to assess the therapeutic potential of manipulating ADH to normalize the osmostat in this population with chronic hyponatremia that remains vulnerable to acute hyponatremia when water loading, driven by primary polydipsia, exceeds their water secretory capacity. Early recognition of a reset osmostat lowers the target level for acute hyponatremia correction and enhances patient safety, since spontaneous or forced correction to a normal serum sodium concentration is potentially dangerous.

## Data Availability

Not applicable.

## Abbreviations

SIADH: Syndrome of inappropriate antidiuretic hormone
Na: Serum sodium concentration
BUN: Blood urea nitrogen
ADH: Antidiuretic hormone
